# The COVID-19 pandemic: implications for the head and neck anesthesiologist

**DOI:** 10.1097/HN9.0000000000000026

**Published:** 2020-05-19

**Authors:** David W. Healy, Benjamin H. Cloyd, Michael J. Brenner, Robbi A. Kupfer, Karina S. Anam, Samuel A. Schechtman

**Affiliations:** Departments of a Anesthesiology; b Otolaryngology-Head and Neck Surgery, University of Michigan Medical School, Ann Arbor, MI

**Keywords:** COVID-19, SARS-CoV-2, Coronavirus disease, Airway management, Head and neck surgery, Personal protective equipment, Viral transmission, Aerosol-generating procedure, High-flow nasal oxygen (THRIVE), Tracheostomy

## Abstract

As the COVID-19 pandemic has unfolded, there has been growing recognition of risks to the anesthesia and surgical teams that require careful consideration to ensure optimal patient care. Airway management and other head and neck procedures risk exposure to mucosal surfaces, secretions, droplets, and aerosols that may harbor the SARS-CoV-2 virus. This review provides guidance on optimal practice approaches for performing patient evaluation and management of head and neck procedures with the shared goal of providing safe and effective patient care while minimizing the risk of viral transmission.

**Methods:**

The scientific literature was evaluated, focusing on strategies to reduce risk to health care workers involved in airway management and head and neck surgery. The search strategy involved curating consensus statements and guidelines relating to COVID-19 or prior coronavirus outbreaks in relation to aerosol-generating procedures (AGPs) and other high-risk procedures, with the search restricted to the scope of head and neck anesthesia. A multidisciplinary team analyzed the findings, using iterative virtual communications through video conference, telephone, email, and shared online documents until consensus was achieved, loosely adapted from the Delphi technique. Items without consensus were so indicated or removed from the manuscript.

**Results:**

Health care worker infection and deaths during the COVID-19 pandemic and prior outbreaks mandate robust standards for infection control. Most head and neck anesthesiology procedures generate aerosols, and algorithms may be modified to mitigate risks. Examples include preoxygenation before induction of anesthesia, rapid sequence induction, closing circuits expeditiously, and consideration of apneic technique for surgical entry of airway. Rescue measures are also modified, with supraglottic airways elevated in the difficult airway algorithm to minimize the need for bag mask ventilation. Personal protective equipment for AGPs include fit-tested N95 mask (or purified air positive respirator), gloves, goggles, and gown for patients with known or suspected COVID-19. Meticulous donning and doffing technique, minimizing personnel and room traffic, diligent hand hygiene, and social distancing all can decrease risks. Perioperative management approaches may differ from commonly employed patterns including avoidance of techniques such as jet ventilation, high-flow nasal oxygen and instead utilizing techniques with a closed ventilatory circuit and secured endotracheal tube, minimizing open suctioning, and preventing aerosolization at emergence. Recommendations are made for the following head and neck procedures and considerations: primary airway management; high-flow nasal oxygen delivery; jet ventilation for laryngotracheal surgery; awake intubation; transnasal skull base surgery; tracheostomy; and use of personal protective equipment.

COVID-19 testing may facilitate decision making, but it is currently often unavailable and urgency of surgical treatment must be considered.

**Conclusions:**

During pandemics, head and neck anesthesia and surgical teams have a duty to not only provide high quality patient care but also to ensure the safety of the health care team. Several specific perioperative approaches are recommended that have some variance from commonly employed practices, focusing on the reduction of AGP to minimize the risk of infection from patients with known or suspected COVID-19 infection.

## Review manuscript body

The COVID-19 pandemic is the most profound “volatility, uncertainty, complexity, and ambiguity” (VUCA)^1^ challenge of the millennium. The emergence of severe acute respiratory syndrome coronavirus^2^ (SARS-CoV-2), a medium-sized RNA virus, in the city of Wuhan, Hubei Province, China has resulted in a disease burden that has overwhelmed health systems and collapsed economies^2^,^3^.

### SARS-CoV-2 virology and clinical implications

SARS-CoV-2 has higher infectivity than prior coronaviruses, including those responsible for Severe Acute Respiratory Syndrome (SARS-CoV-1) and Middle Eastern Respiratory Syndrome (MERS-CoV)^4^. Furthermore, it may be transmitted by infected individuals with mild or no symptoms^5^–^8^. While most cases of COVID-19 are self-limited, the onset of serious disease is often insidious, with a subset of patients who perceive mild symptoms demonstrating significant reductions in oxygenation, portending a precipitous clinical deterioration^9^,^10^.

The combination of rapid transmission and an incidence of severe disease requiring early critical care support (including mechanical ventilation) has strained many health systems and providers during the course of the pandemic. Resource scarcity has exacerbated these challenges with pervasive shortages of the personal protective equipment (PPE) necessary for safety of frontline health care workers.

Transmission of the SARS-CoV-2 virus occurs by droplet spread (large particles >5 μm produced by breathing, coughing, or sneezing) and by direct contact with contaminated inanimate objects (fomites). Droplet spread has a limited range for transmission (<1 m) given the propensity of the droplets to fall out of the air from size and gravity. The virus can also be transmitted by aerosolization, with minute particles (<5 μm) suspended in the air^11^,^12^. Experimental data document that aerosolized SARS-CoV-2 remains viable for 3 hours and virus on plastic or steel may remain viable at 72 hours^11^.

Although guidelines exist for intubation and bag-mask ventilation, less has been written about head and neck surgical procedures, many of which can generate aerosolized virus^13^. Reports indicate that health care teams participating in these procedures are at increased risk of infection^14^. This article describes the anesthetic and surgical care of patients undergoing head and neck procedures during the COVID-19 pandemic. We present recommendations for scheduling, procedure selection, airway management, PPE, and preprocedural testing.

An iterative approach, adapted from Delphi methodology, was used to prioritize topics for inclusion and to reach consensus on recommendations. The first round consisted of drafting distinct statements for the inclusion of this manuscript, making recommendations on case selection, timing, performance, and management of procedures, based on the primary data sources retrieved. These recommendations were circulated among all authors, inviting both opinion and any additional recommendations. The second round of review was conducted incorporating new recommendations, along with those recommendations from the first round that had received a favorable comment. A final review of the recommendations was made during the final stage of drafting, and a videoconference was conducted, followed up with electronic correspondence and telephone discussions, facilitating refinement to the content of this manuscript.

### Airway management considerations

The concept of aerosol-generating procedures (AGPs) emerged during the 2002–2004 SARS-CoV-1 outbreak (SARS outbreak). Aerosolized viral particles, distinct from droplets and fomites, may become airborne during a variety of commonly performed airway management procedures^13^. The infection of health care workers during the SARS outbreak led to illness and deaths that secondarily impaired patient care^14^. During the COVID-19 outbreak, the Designated Hospital of Wuhan, China showed increased nosocomial SARS-CoV-2 infection in workers exposed to aerosolizing procedures^15^. As of April 1, 2020, 388 health care worker deaths from COVID-19 had been documented^16^ underscoring the need for ensuring the safety of clinicians.

**Figure 1:**
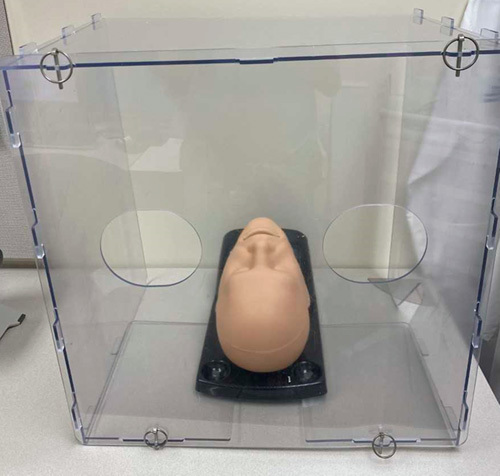
Aerosolization barrier device: AeroGuard, Schupan Aluminum and Plastic Sales (Kalamazoo, MI).

Recent evidence supports the theory that SARS-CoV-2 is transmitted via aerosolization of virus particles^17^, posing a risk of infection during airway management procedures and other head and neck surgical procedures. Recommendations exist for general airway management procedures (including tracheal intubation, bag-mask ventilation, and laryngeal mask insertion) in patients suspected of, or infected with SAR-CoV-2^18^,^19^ (**Table 1**). Several of these recommendations also highlight the importance of teamwork, preparation, communication, and safety verifications in addition to procedural approaches^20^. Use of novel devices for airway management, such as barrier enclosures (ie, boxes, drapes) may help to limit aerosolization during intubation, extubation, and/or with use of oxygen delivery devices (**Fig. 1**). Yet they still remain unproven and may introduce challenges, such as limited range of motion or impaired visualization^21^,^22^. Further investigation is needed to define the role of such devices.

**Table 1:**
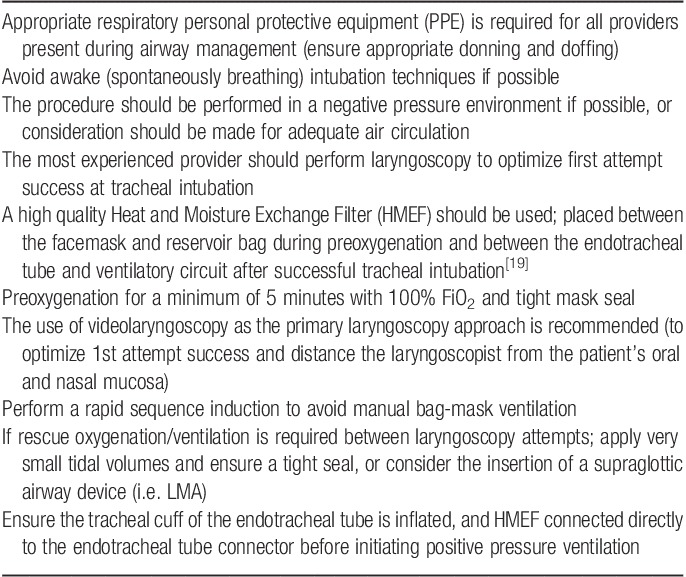
COVID-19 airway management considerations and recommendations

Certain procedures warrant special attention, due to their predilection for generating infectious aerosols. These procedures include awake flexible endoscopic intubation; use of high-frequency jet ventilation; transnasal humidified rapid insufflation ventilatory exchange (THRIVE); and high-flow nasal oxygenation (HFNO). Although data are limited on the magnitude of aerosol generation associated with various procedures, experimental studies document that procedures that generate air currents with aerosol are conducive to wider spread^23^. A variety of AGPs may be encountered during patient care including multiple heads and neck operative, diagnostic, and therapeutic procedures (**Table 2**)^18^,^23^–^25^.

**Table 2:**
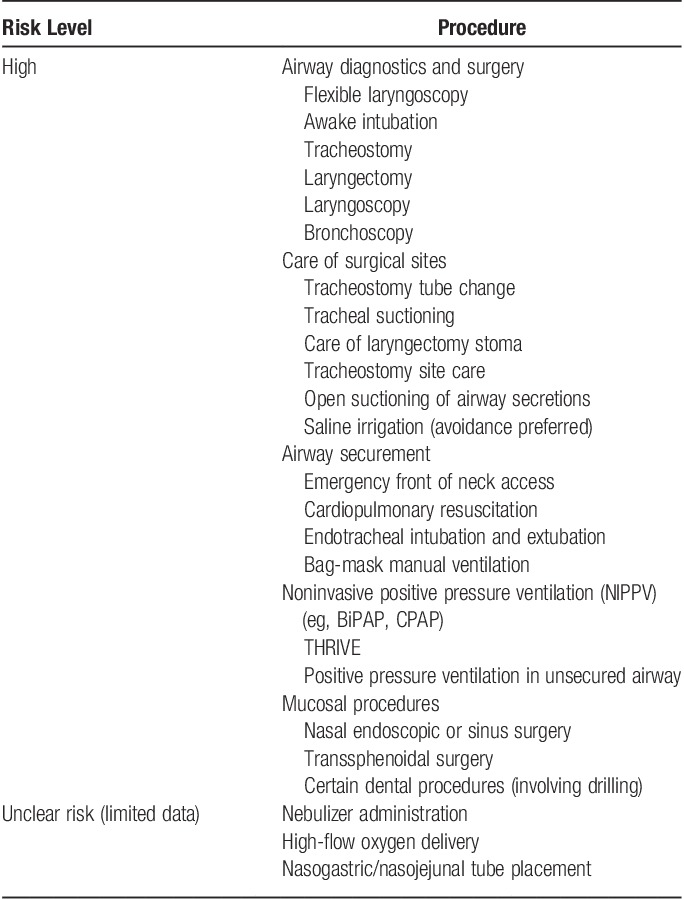
Classification of the risk of aerosol-generating procedures

### Awake flexible endoscopic intubation

Traditional awake flexible endoscopic (fiberoptic) intubation is considered an AGP and therefore requires careful risk assessment and mitigation before it is performed. If a nebulized solution of local anesthetic is used for topicalization, this can theoretically cause aerosolization and may compound the risk of infection to health care providers^26^. The perceived risk of nebulizers is not universally accepted, and previous studies performed during the SARS-CoV-1 outbreak did not link the use of nebulizers to the transmission of infection^23^,^27^. Nonetheless, the procedure can cause gagging and coughing, which increases the risk of the droplet and aerosol spread. Flexible laryngoscopy by surgical providers is similarly considered to be a high risk^28^. Given these risks, current guidance from the Anesthesia Patient Safety Foundation recommends avoiding awake endoscopic (fiberoptic) intubation in patients with suspected coronavirus (SAR-CoV-2) infection, unless specifically indicated^19^.

There are some instances where an awake intubation seems difficult to avoid. For example, a patient with an anatomically abnormal airway requiring airway support for oxygenation/ventilation may still require awake intubation. If the decision is made to proceed with awake intubation, the procedure is ideally performed in an airborne isolation negative pressure room. Full respiratory PPE should be worn by each member of the health care team present in the room, and the individuals in the room should be minimized.

**Figure 2:**
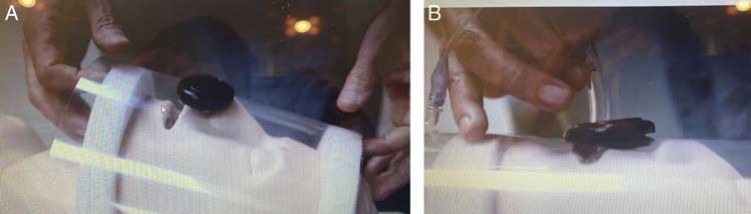
Novel Face Shield Akervall Technologies Inc. (Saline, MI). A, Novel Face Shield used to limit aerosolization during flexible nasal endoscopy and/or awake nasal flexible endoscopic intubation. B, Novel Face Shield being used to limit aerosolization when performing awake oral flexible endoscopic intubation.

Given the concern for potential viral transmission, the use of nebulized local anesthetic for topicalization is discouraged^26^. The use of intravenous low dose potent opioid (eg, remifentanil infusion) may be useful to reduce coughing during airway topicalization and should be considered with the patient under full monitoring and direct vision of the anesthesia provider. The “swish and swallow” technique is unlikely to lead to coughing or aerosolization, as is judiciously delivered local anesthesia via an atomizer directly onto mucosa. Regional techniques may also be considered although these too can cause patient reaction with coughing and viral spread. Use of other protective devices such as an endoscopy Faceshield (**Fig. 2**) may also limit virus contamination during endoscopic intubation (Akervall Technologies Inc., Saline, MI).

In patients with a difficult airway who require tracheostomy, the surgical procedure is often preceded by awake fiberoptic intubation, with induction and maintenance of anesthesia to facilitate the surgical procedure. With this approach, there is a risk of viral aerosolization during both the fiberoptic intubation and the surgical tracheostomy, consideration should be made for the performance of awake tracheostomy under local anesthesia alone to reduce the risk of viral aerosolization and infection of the health care team. This would result in a single aerosolization procedure being performed, instead of two. Awake tracheostomy presents a high risk of aerosolization and should be performed with caution, using surgical and anesthetic techniques that minimize aerosol generation^29^.

### Jet ventilation

High-frequency jet ventilation is often utilized during laryngoscopic surgery for diagnosis and treatment of conditions such as subglottic stenosis and posterior glottic stenosis. High-frequency jet ventilation can also be delivered through a cannula cricothyroidotomy in emergency front of neck access^30^,^31^.

During the SARS-CoV-1 outbreak in 2003, the use of jet ventilation in a patient presenting with respiratory infection resulted in a super spreading event linked to 138 infections, many of whom were health care workers^13^. Given its characteristic high frequency and pressure, jet ventilation entails a high risk of aerosolization and the dissemination of viruses when used during open airway procedures. During the pandemic, we recommend against jet ventilation for rescue in a cannot intubate, cannot oxygenate event. In such instances, a scalpel bougie technique is preferred to aerosolization without a secured cuffed tube. Also for laryngotracheal procedures, consideration should be made for intermittent apnea techniques that permit mechanical ventilation through a closed airway circuit. This approach uses a secured airway with ventilation to minimize the risk of aerosolization and is supported by the American Academy of Otolaryngology—Head and Neck Surgery^32^.

### HFNO

There remains significant debate regarding whether the use of HFNO including THRIVE is appropriate in patients with suspected or proven COVID-19 disease^33^,^34^. Several sources recommend against the use of HFNO until evidence can establish its safety^35^–^37^. Nonetheless, HFNO has been utilized in many intensive care units, based on the premise that it may obviate the need for intubation in some patients; this stance continues to be debated^33^,^38^,^39^. If HFNO is used, the patient should ideally be placed in a negative pressure environment; although devices that limit aerosolization or distribution are also an option^40^. Prior investigation with HFNO in simulation did not find it to increase the risk of droplet and contact infection^34^, however, in the operating room, intermittent apnea should be utilized over apneic oxygenation via HFNO whenever possible. If HFNO is utilized, facemask protection should be placed on the patient to limit aerosolization. In patients with COVID-19 and severe respiratory compromise, the use of HFNO to maintain apneic oxygenation is unlikely to be as effective as in healthy patients^41^,^42^.

### General considerations for head and neck surgery

Deferring nonurgent cases has many benefits, despite the delay in care. Deferring cases decreases high-risk physical interactions amongst individuals, preserves PPE to protect health care workers, and helps to ensure the availability of equipment and facilities that can be repurposed to provide in-patient and critical care of patients suffering from COVID-19. On March 24th, a joint statement from the American College of Surgeons, American Society of Anesthesiologists, and the Association of periOperative Registered Nurses recommended the use of triage criteria^43^, with oversight and decision making by a Surgical Review Committee of all operations^44^.

The American Academy of Otolaryngology–Head and Neck Surgery emphasizes that SARS-CoV-2 virus particles reside in high concentrations within the nasopharynx and within oropharyngeal and nasopharyngeal secretions (AAO-HNS Position Statement). Otolaryngologists, oral and maxillofacial surgeons, dentists, and anesthesiologists are susceptible to viral transmission through mucus, blood, and aerosolized particles when examining or performing procedures in these anatomical locations^45^–^48^. From the COVID-19 experience in Wuhan, China, several of the physicians who became infected and even died were anesthesiologists, critical care providers, ophthalmologists, and otolaryngologists^46^.

In the SARS-CoV-1 and MERS-CoV epidemics, most cases of infection involved transmission within health care systems that were associated with AGPs^19^. According to the American Academy, evidence from China, Italy, and Iran during the current COVID-19 pandemic show that otolaryngologists are among the highest risk for contracting the virus while performing upper airway procedures, if not using appropriate PPE^35^,^46^,^47^,^49^,^50^ (**Table 3**).

**Table 3:**
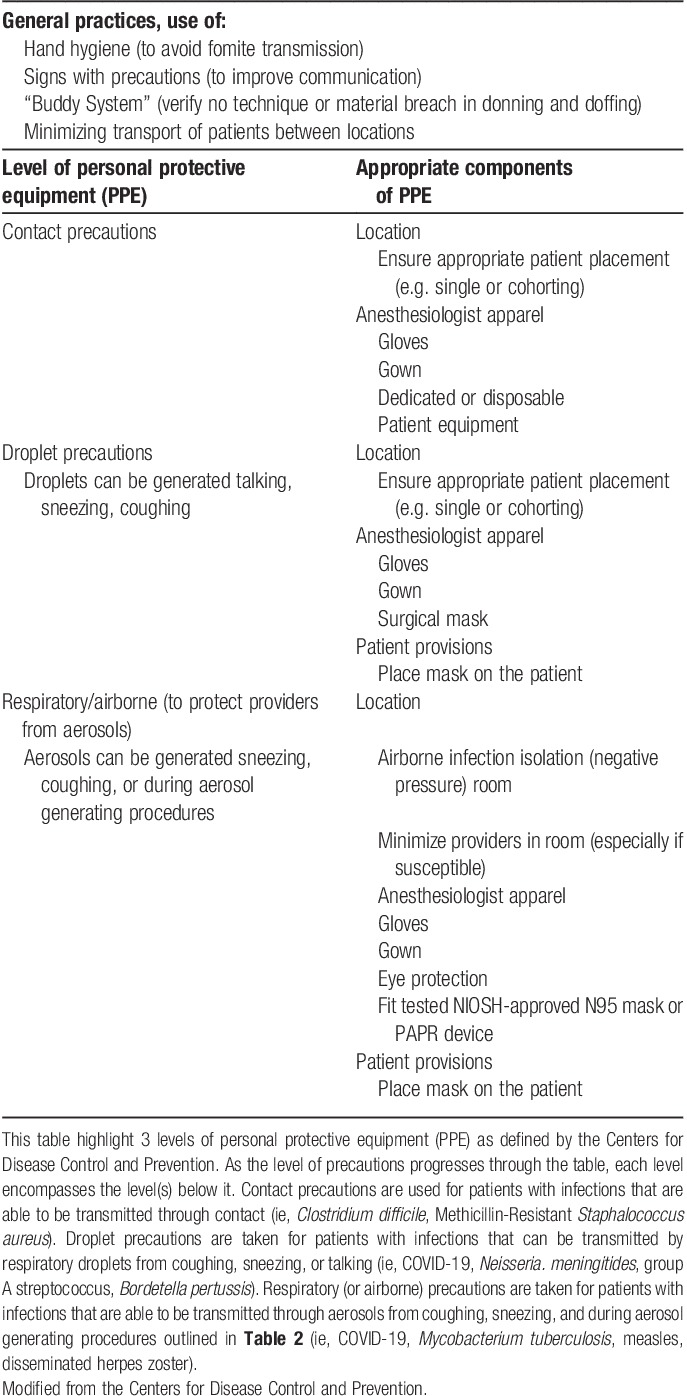
Infection control considerations and personal protective equipment

Airway surgery is inherently aerosol-generating, and many patients present without signs or symptoms of infection^13^. The recent position statement from the American Academy of Otolaryngology and Head and Neck Surgery states that if COVID-19 status of the patient cannot be confirmed, then the patient should be handled as if they are COVID-19 positive^49^. This position is supported by other discussion and commentary^47^,^48^. Teamwork and communication between anesthesiology and the head and neck surgical teams is vital for procedural planning. These discussions should consider the risk of transmission to other patients and to health care providers, as well as the benefits of stewarding PPE if procedures can be deferred. Possible strategies include: rescheduling, relocating, and the use of correct standards of PPE.

### Transnasal skull base surgery recommendations

The SARS-CoV-2 virus maintains high concentrations in the nasopharynx and nasal secretions^51^; endoscopic transnasal surgery, and transsphenoidal surgery has a high risk for transmitting disease. From reports in China, transsphenoidal pituitary surgery resulted in multiple health care providers becoming infected and requiring quarantine often despite using appropriate respiratory PPE including N95 masks^46^. On the basis of these presented cases and reviewed reports, the American Academy of Otolaryngology—Head and Neck Surgery (AAO-HNS reference) recommended that endoscopic transnasal surgery should only be performed for urgent/emergent needs. If preoperative COVID-19 testing is performed and the patient is found to be positive, surgery should be delayed until the infection is cleared. In addition, given the possibility of false negatives and risk with surgery, respiratory PPE should universally be utilized for these cases.

### Tracheostomy recommendations

The American Academy of Otolaryngology—Head and Neck Surgery has developed tracheostomy recommendations during the COVID-19 Pandemic^52^. As an AGP, tracheostomy timing aims to minimize risk of viral exposure by considering timing, procedural approaches, and use of PPE. Tracheostomy should not be performed in patients with unstable pulmonary status nor sooner than 10–21 days weeks from initial intubation, preferably once the viral load has decreased (viral RNA can sometimes be detected by PCR for up to 20 days from symptom onset; however, the immune response is often activated and this virus is typically culture negative at these later time points). In addition, clinicians should bear in mind that days intubated will usually underestimate the duration elapsed since infection^45^. Still, there is a lack of high-quality data on the degree of aerosolization with open versus percutaneous tracheostomy techniques. Several recommendations are considered important to optimize success and patient and provider safety during tracheostomy procedures (**Table 4**)^52^.

**Table 4:**
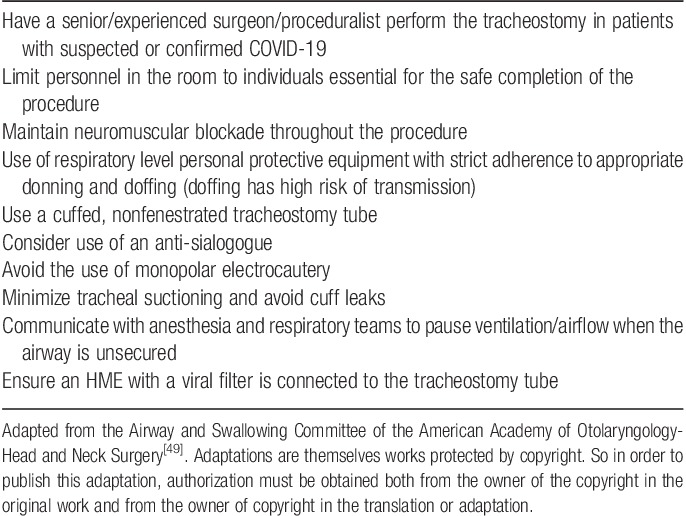
Considerations for performing a tracheostomy during the COVID-19 pandemic

### PPE

For AGP, airborne precautions include the use of gowns, gloves (often inner and outer layer recommended), eye protection and/or shield, and an N95 mask or purified air positive respirator (PAPR). Airway procedures also present a significant risk of transmission in patients with undiagnosed disease who are asymptomatic.

Whether an N95 or PAPR provides superior protection in AGP represents an ongoing debate. In a review of the 2009 Influenza A Pandemic (H1N1), the CDC recommended that providers involved in AGP wear at a minimum an N95 respirator and may consider a powered air-purifying system (PAPR). The PAPR was shown to provide 2.5–100 times greater protection than the N95^53^. The N95 efficacy is limited by the potential for face seal leakage, which is not always predicted by fit testing. Following the SARS-CoV-1 outbreaks in 2003, it was found that 9% of health care workers that intubated patients contracted SARS^54^, suggesting that aggressive measures were required to protect health care workers from the viral transmission. The PAPR system is not without drawbacks as it requires appropriate assembly, maintenance, donning, doffing, and handling once it enters into the care setting and becomes contaminated. It may also limit hearing and communication, interfering with procedures. Regardless of the choice of respirator used, appropriate training and handling to prevent contamination are key to optimize its efficacy.

### Preprocedural testing

As the COVID-19 pandemic evolves, scientific understanding of the spread of the disease improves. New evidence has demonstrated that many infected individuals may have either no or very mild symptoms^55^–^58^, and asymptomatic shedding is likely a common form of viral transmission^59^,^60^. When preparing patients for surgery it is important to understand that viral respiratory shedding is thought to be greatest after the first week of infection, and may precede obvious signs or symptoms^59^.

Diagnosis is made by the detection of viral RNA, typically using a reverse transcriptase-polymerase chain reaction (RT-PCR)^60^. Nasopharyngeal swabs have demonstrated greater sensitivity than oropharyngeal swabs^59^, and as a single negative test may be misleading, some recommend repeated testing after a single negative test^59^. In addition to diagnostic tests identifying the presence of the virus, serological tests exist to measure the number of antibodies or proteins present in the blood when the body is responding to COVID-19. Antibody tests have been positive in 90% of infected individuals by 11–24 days after infection^61^,^62^. While limitations exist to these tests’ effectiveness for diagnosing prior COVID-19 infection, these serological tests can play a critical role in the fight against COVID-19 by helping health care professionals identify individuals who have overcome infection and developed an immune response. Despite improvements in serological detection, a further consideration is that the degree and duration of protection afforded through immunity after infection with SARS-CoV-2 remain uncertain^56^,^63^. While COVID-19 infection remains prevalent, testing of both symptomatic and asymptomatic individuals is advisable before proceeding with head and neck surgical procedures. Timely and accurate testing results allow perioperative patient outcomes to be optimized, and it also may help ensure the safety of health care workers and ensure appropriate stewardship of scarce PPE.

## Conclusions

SARS-CoV-2 has rapidly spread across the world, and preliminary evidence supports COVID-19 having a similar pattern of transmission as SARS-CoV-1 and MERS-CoV via droplets, fomites, and respiratory aerosols.

The operative care of patients undergoing head and neck procedures involves an increased risk of viral transmission during both the surgical procedures themselves and the anesthesia techniques required to provide optimal care. Each case requires team-based communication and collaboration to ensure complete understanding and evaluation of the risk and benefits of preoperative preparation, the timing of care, the proposed surgical procedure, and the required anesthesia techniques (**Table 5**). With the continued widespread prevalence of SARS-CoV-2 infection in our communities (with the symptomatic and asymptomatic disease) the use of accurate and timely preoperative testing will prove vital to optimize perioperative workflow and management. As we move into the next stages of the pandemic, the management of head and neck procedures will require a determined collaborative effort to help patients while continuing to protect health care providers.

**Table 5:**
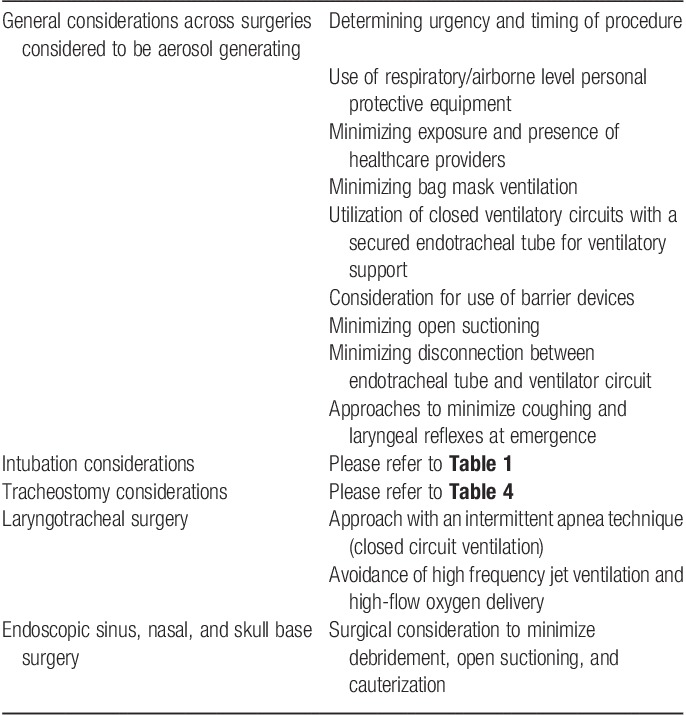
Summary of COVID-19 considerations for head and neck surgery

## Authors’ contribution

D.W.H., B.H.C., M.J.B., R.K, S.A.S..: Literature review, conceptual design, drafting, and review of the manuscript. K.S.A.: Literature review, drafting, and review of the manuscript.

## Conflict of interest disclosures

The authors declare that they have no financial conflict of interest with regard to the content of this report.
